# Influence of dosimetry accuracy on the correlation with treatment outcome in a preliminary PSMA radiopharmaceutical therapy study

**DOI:** 10.1007/s00259-024-07010-3

**Published:** 2024-12-20

**Authors:** Jiaxi Hu, Robert Seifert, Sofia Karkampouna, Carlos Vinicius Gomes, Song Xue, Ali Afshar-Ormieh, Axel Rominger, Kuangyu Shi

**Affiliations:** 1 Department of Nuclear Medicine, Inselspital, Bern University Hospital, University of Bern, Freiburgstrasse 20, 3010 Bern, Switzerland; 2 Urology Research Laboratory, Department for BioMedical Research, University of Bern, Bern, Switzerland; 3 Department of Urology, Inselspital, Bern University Hospital, University of Bern, Bern, Switzerland; 4 Department of Biomedical Imaging and Image-Guided Therapy, Division of Nuclear Medicine, Medical University of Vienna, Vienna, Austria

**Keywords:** ^177^Lu-PSMA-617, Dosimetry, Prostate cancer, Theranostics, Radionuclide therapy

## Abstract

**Introduction:**

Despite the potential of dosimetry in optimizing personalized radiopharmaceutical therapy (RPT), its limited clinical implementation impedes the development of simplified protocols for routine adoption. However, simplifications may introduce errors in dosimetry, prompting questions about their impact on clinical practice.

**Materials and methods:**

In this retrospective study, we analyzed data from 21 patients diagnosed with metastatic castration-resistant prostate cancer (mCRPC) who underwent multiple cycles of ^177^Lu-PSMA-617 RPT treatment. Cumulative dosimetry of all the treatment cycles was calculated using both the standard multi-time point dosimetry (MTPD) method and the single time-point dosimetry (STPD, Hänscheid approximation) method for the same cohort. Their correlations with treatment outcome (PSA decline rate and overall survival, OS) and complication risk (anaemia grade) were investigated. The Fisher's Z-Transformed test was performed to statistically evaluate the difference between the correlations.

**Results:**

STPD showed a non-significant difference in correlation with PSA decline rate, despite a mean percentage error (MPE) of up to 36.44% in tumor dosimetry compared to MTPD (MTPD: rho = -0.39, *p* < 0.001; STPD: rho = -0.46, *p* < 0.001; Z = 0.58, *p* = 0.56). Both STPD_total_ and MTPD_total_ demonstrated a significant impact on OS (STPD_total_: Hazard Ratio = 1.05, *p* < 0.05, log-transformed MTPD_total_: Hazard Ratio = 3.41, *p* < 0.05, log-transformed STPD_total_: Hazard Ratio = 8.06, *p* < 0.05). Additionally, despite a MPE of up to -40.26% in bone marrow dosimetry, STPD showed a non-significant difference in correlation with anemia grade (MTPD: rho = 0.35, *p* < 0.001; STPD: rho = 0.40, *p* < 0.001; Z = -0.39, *p* = 0.70).

**Conclusion:**

The preliminary findings from a small cohort indicate that the reduced accuracy of a clinically simplified protocol may not diminish the clinical therapy outcome predictive value of dosimetry. Future thorough systematic investigations may be needed to determine the clinically acceptable level of accuracy for dosimetry.

**Supplementary Information:**

The online version contains supplementary material available at 10.1007/s00259-024-07010-3.

## Introduction

Prostate cancer (PCa) is a leading cause of cancer-related deaths in men globally [1]. In particular, metastatic castration-resistant prostate cancer (mCRPC) is challenging to treat and ultimately leads to death [2]. Over the past decades, treatment strategies for mCRPC have expanded to PSMA-directed radiopharmaceutical therapy (PSMA-RPT), which has shown promising efficacy and survival benefits [3, 4]. The emerging advancement of PSMA-RPT has placed significant emphasis on the role of dosimetry for personalized treatment [1–7]. Dosimetry-based treatment planning has been advocated by European Council Directive 2013/59/EURATOM, emphasizing dosimetry via Multiple-Time-Point Dosimetry (MTPD) to ensure accurate clearance measurements [8–10].

Recent studies have explored diverse dosimetry methods to enhance precision in therapeutic radiopharmaceutical applications. For example, innovative approaches include 3D Monte Carlo-based voxel-wise dosimetry accounting for tissue compositions [11, 12], quantitative calibration incorporating multiple energy windows for scatter correction during reconstruction [13, 14], and the integration of AI algorithms to refine patient-specific internal dosimetry through advanced segmentation methods and pre-therapeutic PET scan-based predictive models [15, 16]. These advancements highlight a multifaceted approach to dosimetry optimization.

Despite these advancements, challenges persist in clinical implementation, including workflow constraints, patient tolerance, and resource limitations [17–19]. To address these challenges, studies have explored alternative dosimetry strategies like Single-Time-Point Dosimetry (STPD), demonstrating comparability with MTPD in previous research [20, 21]. STPD offers a simplified approach potentially reducing clinical burden, yet comprehensive clinical evaluation in ^177^Lu-PSMA-617 RPT therapy remains limited.

Correlation studies have highlighted the impact of dosimetry on treatment efficacy, linking cumulative administered activity to overall survival (OS), and revealing significant differences in PSA response groups using MTPD-based tumor absorbed doses [22–24]. Notably, initial studies have also validated the comparability of the simplified dosimetry approach in monitoring hematologic safety [25]. However, there remains a gap in our understanding of the impact of cumulative absorbed doses in tumor from a therapy outcome-driven perspective, as well as in the evaluation of error tolerance in dosimetry methods.

This study aims to assess the error tolerance of STPD compared to MTPD and characterize patient-specific dosimetry from a clinical perspective, quantifying whole-body tumor and organs at risk (OARs) doses over cycles. It investigates the influence of dosimetry errors in STPD relative to MTPD and their correlations with clinical outcomes and toxicity grading. This research will contribute evidence-based recommendations for dosimetry evaluation in therapy management.

## Materials and methods

### Patients characteristics

Cantonal Research Ethics Committee review board approved our study, and all patients provided written informed consent (Ethical approval numbers: 2023–02053, 2023-01877). We retrospectively included 21 patients with mCRPC, who received ^177^Lu-PSMA-617 RPT between 10.2019 and 12.2022. PSMA-positive disease was confirmed via pre-therapeutic ^68^ Ga- or ^18^F-PSMA-PET/CT. Serum PSA values were recorded longitudinally for each therapy cycle for each individual. Quantitative SPECT/CT scans at 2–4 h, and time-points among 1st-9th days post injection (p.i.) were collected for dosimetry evaluation, totaling at least 3 time-points.

### Dose estimation

We employed the VoxelDosimetry tool from Hermes Medical Solutions (https://www.hermesmedicalsolutions.com/voxel-dosimetry/) to generate MTPD dose-maps, and STPD dose-maps were generated using the built-in Hänscheid approximation [26]. Segmentations were semi- or automatically delineated from pre-treatment ^68^ Ga- and ^18^F-PSMA-PET. Multiple normal organs segmentations, including liver, kidneys, spleen, and whole-body bones were obtained using a CT-based deep learning method (Multiple-organ objective segmentation, MOOSE [27]). The non-tumor infiltrated bone marrow segment was computed using whole-body bones mask from MOOSE, subtracted by PSMA accumulation showing above 50% whole-body maximum standard uptake value (SUV_max_ > 50%). PSMA-positive whole-body tumor segmentation was computed semi-automatically by iso-contouring based on patient-specific global thresholding [28]. Volumes-of-interest (VOIs) were drawn and registered using PMOD software (PMOD Technologies Ltd., Zurich, Switzerland). Organ-wise effective half-life (*T*_*eff*_) was approximated using a bi-exponential decay fitting model with multiple time-points of SPECT concentrations obtained after administration [29]. To narrow down Hänscheid STPD error, only the STPD dose-maps’s scan time-points in a range of 0.75 * *T*_*eff*_ < time-points < 2.5 * *T*_*eff*_ were considered for dose measurement [26, 30]. All MTPD and STPD dose-maps were used to extract mean physical absorbed doses, and cumulative doses were computed over cycles for further analysis, referring to MTPD and STPD (in Gy), respectively. Accordingly, total absorbed doses were calculated for individuals, referring to MTPD_total_ and STPD_total_ (in Gy).

### Treatment response assessment

The overall primary therapy outcome of interest was OS, defined as the survival time after the first cycle administration day. Secondary treatment response was demonstrated biochemically by serum PSA, classified according to Prostate Cancer Working Group 3 (PCWG3). Serum PSA tests were performed 1 day before and 3–4 days after each ^177^Lu-PSMA-617 RPT administration. PSA decline rate was calculated using the formula: (PSA at the end of each cycle or 1 day before administration for next cycle minus PSA at baseline)/PSA at baseline. The good response event was defined as post-therapeutically confirmed PSA decrease ≥ 50% compared to the baseline. The incidence of treatment-emergent adverse events (TEAEs) [31] was documented and classified by Common Terminology Criteria for Adverse Events (CTCAE v5.0) [32]. Safety and tolerability represented by the rate of grade ≥ 3 events, which were also documented for individual.

### Statistical analysis

Frequency analyses, descriptive statistics, statistical comparisons, Cox regression were carried out using SPSS software (IBM SPSS Statistics 28.0, New York). Correlation between dosimetry parameters and therapy outcome indicators (PSA decline rate) was evaluated using the Spearman correlation test. The Fisher's Z-Transformed test was utilized to statistically assess the differences in correlations between the two dosimetry methods and the clinical parameters mentioned above. Univariate Cox regression analyse was utilized to assess the association of whole-body tumor STPD_total_ and MTPD_total_ on the overall response factor (OS). A two-sided *P*-value < 0.05 was considered significant.

## Results

### Patient characteristics

All 20 patients (age range: 60–84 years old) received 84 cycles of ^177^Lu-PSMA-RPT therapy, involving 206 single-time-points of SPECT/CT scans (scans on Day 1 p.i.: *n* = 77, Day 2 p.i.: *n* = 81, Day 3 p.i.: *n* = 48). One patient was excluded from the dosimetry estimation phase due to only having dosimetry data from the 4th cycle. The time interval between two treatment cycles was 5–8 weeks.

### Treatment response and toxicity

PSA response is depicted in a waterfall plot (Supplemental Fig. 1) for the 1st-6th cycles. Any PSA decreased response was seen in 18 patients after the first cycle, and 13 patients demonstrated a greater than 50% decline in PSA compared to baseline during their therapy. Median OS was approximately 15 months p.i. (range: 53–1128 days p.i.) in this cohort. ^177^Lu-PSMA-617 RPT was well tolerated by all patients. Treatment-emergent grade 1 (*n* = 5), 2 (*n* = 13), and 3 (*n* = 1) anaemia were reported during hospitalization. As for non-hematological toxicity, transient xerostomia (Grade 1) was reported in 4 patients, and 3 patients experienced declined eGFR (Grade 2). Discrete taste disturbances and mild xerophthalmia were observed in 1 patient. Grade 4 or higher adverse effects were not observed in any of the patients.

### Dosimetry estimation

The population-based *T*_*eff*_ for whole-body tumor was approximated as 45.05 h. *T*_*eff*_ for OARs were approximated as follows: 29.86 h for bone marrow, 35.11 h for kidneys, and 38.40 h for liver, 34.02 h for spleen. Therefore, only STPD-based dose-maps obtained during Day 1–3 p.i. were included for further analysis. STPD and MTPD (in Gy) across the 1st–6th therapy cycles were computed for 20 patients. The corresponding descriptive statistics are summarized in Table 1.

**Table 1:** Descriptive statistic of MTPD and STPD (in Gy) across cycles in Tumor, and OARs, including kidneys, liver, spleen, and bone marrow

Cycle	Cumulative Injection Activity (GBq)	Doses (Gy)	Kidneys	Liver	Bone marrow	Spleen	Tumor
1st	7.19 ± 0.28	MTPD	2.59 ± 0.96	0.90 ± 0.50	3.23 ± 3.84	0.63 ± 0.49	14.33 ± 11.55
		STPD	2.08 ± 0.86	0.65 ± 0.56	1.82 ± 2.02	0.39 ± 0.37	14.32 ± 10.74
2nd	14.12 ± 0.59	MTPD	5.65 ± 2.39	1.76 ± 0.83	5.46 ± 6.22	1.18 ± 0.55	25.10 ± 21.25
		STPD	4.70 ± 1.90	1.26 ± 0.71	3.36 ± 3.54	0.74 ± 0.46	23.40 ± 15.41
3rd	21.14 ± 0.80	MTPD	9.10 ± 3.75	2.74 ± 1.20	7.06 ± 9.06	1.79 ± 0.75	31.61 ± 27.07
		STPD	7.61 ± 3.34	2.05 ± 0.96	4.57 ± 5.74	1.23 ± 0.69	29.53 ± 19.66
4th	27.93 ± 1.19	MTPD	12.18 ± 3.94	3.69 ± 1.57	8.12 ± 10.47	2.53 ± 0.90	35.65 ± 29.19
		STPD	10.81 ± 4.20	2.80 ± 1.19	5.43 ± 6.96	1.72 ± 0.77	35.79 ± 21.26
5th	34.83 ± 1.63	MTPD	14.82 ± 4.73	3.97 ± 1.09	9.62 ± 12.11	3.17 ± 1.10	38.30 ± 32.79
		STPD	13.86 ± 5.14	3.11 ± 0.88	6.26 ± 8.42	2.07 ± 0.80	38.61 ± 24.28
6th	39.91 ± 1.42	MTPD	17.20 ± 6.58	4.84 ± 1.36	9.43 ± 9.94	3.71 ± 1.20	32.54 ± 12.70
		STPD	16.17 ± 7.25	3.80 ± 1.06	5.47 ± 5.14	2.64 ± 0.82	37.35 ± 20.22

### Comparison of MTPD and STPD

#### MTPD and STPD per cycle

In the first cycle, tumor MTPD and STPD per unit activity were 2.02 ± 1.68 Gy/GBq and 2.00 ± 1.54 Gy/GBq, respectively. MTPD and STPD per unit activity were highest in the bone marrow (MTPD: 0.45 ± 0.53 Gy/GBq, STPD: 0.25 ± 0.27 Gy/GBq) and kidneys (MTPD: 0.36 ± 0.13 Gy/GBq, STPD: 0.29 ± 0.12 Gy/GBq) among the OARs, followed by the liver (MTPD: 0.12 ± 0.07 Gy/GBq, STPD: 0.09 ± 0.08 Gy/GBq), and spleen (MTPD: 0.09 ± 0.07 Gy/GBq, STPD: 0.05 ± 0.05 Gy/GBq). In cycles 2–6, MTPD and STPD per unit activity per cycle among OARs were similar to those in cycle 1, except for a decrease in the values documented for bone marrow and tumors over the cycles, as summarized in Supplemental Table 1.

#### Correlation of MTPD and STPD

Spearman correlation validated the strong agreement between MTPD and STPD for tumor areas/regions (rho = 0.82, *p* < 0.001), and all studied OARs (bone marrow: rho = 0.94, *p* < 0.001; liver: rho = 0.97, *p* < 0.001; kidneys: rho = 0.96, *p* < 0.001; spleen: rho = 0.95, *p* < 0.001) (Supplemental Fig. 2).

**Fig. 1:**
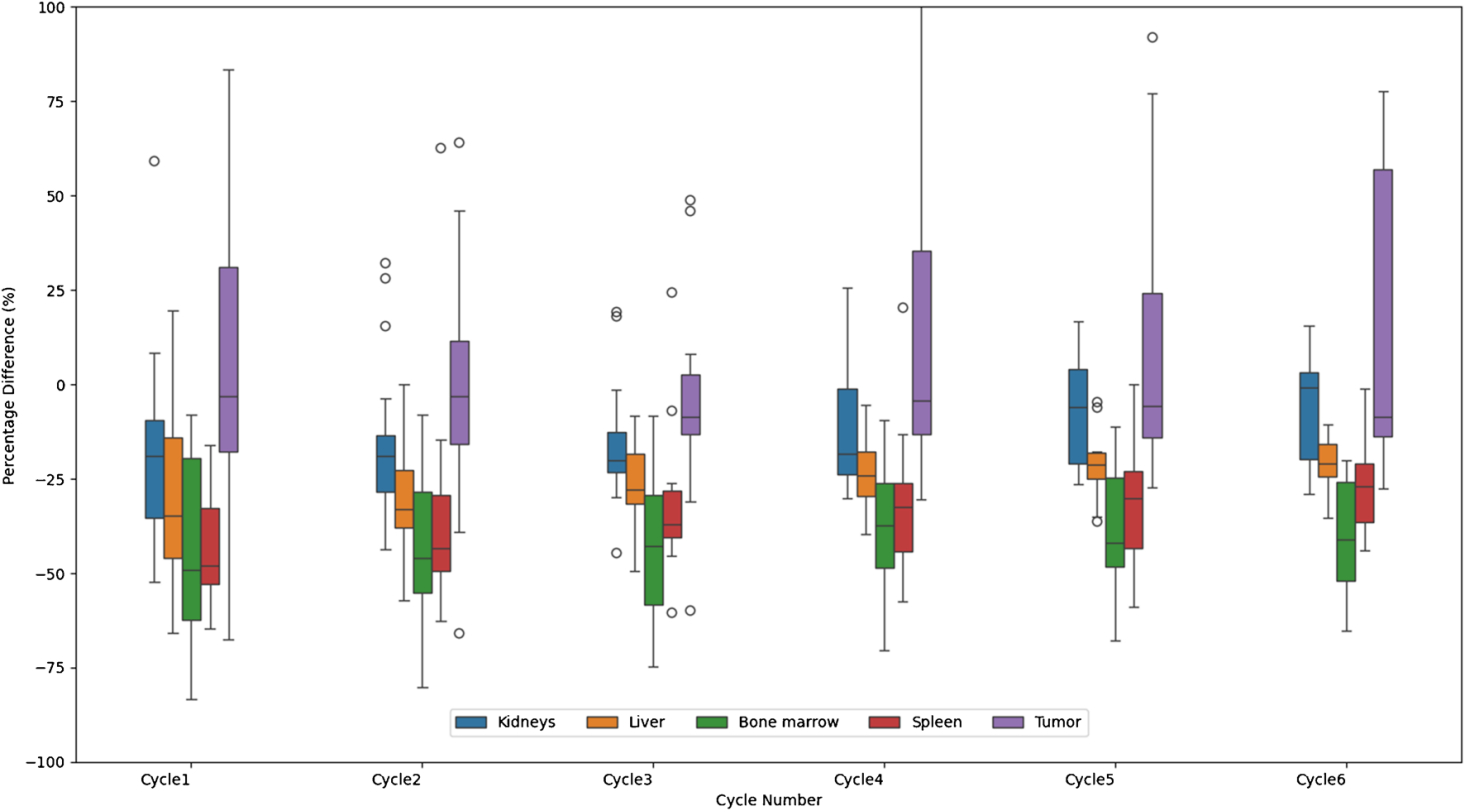
Error bar (Percentage difference, %) of STPD versus MTPD over cycles in tumor and OARs

Errors between MTPD and STPD across all cycles were assessed by Mean Percentage Error (MPE), Relative Percentage Difference (RPD), and Root Mean Squared Error (RMSE). These metrics are summarized in Table 2*.* In Fig. 1, percentage differences (PD) over cycles showed that STPD were generally lower than MTPD for tumor and OARs. The RMSE showed an increasing trend over cycles for all observed organs, while RPD showed a declining trend over cycles for the kidneys, liver, and spleen. The maximum MPE was found in the first cycle for the kidneys (MPE = −18.49%), liver (MPE = −31.78%), spleen (MPE = −30.55%). For the bone marrow and tumor, the maximum MPE was observed in the last cycle (Bone marrow: MPE = −40.26%, tumor: MPE = 36.44%).

**Table 2:** Compare the MTPD and STPD (in Gy) in Tumor, and OARs, including kidneys, liver, spleen, and bone marrow using RMSE, MPE and RPD

Organs	CycleN	RMSE	MPE	RPD
Kidneys	Cycle1	0.81	−18.49	−23.85
	Cycle2	1.51	−14.66	−18.29
	Cycle3	2.05	−15.83	−18.80
	Cycle4	2.35	−11.31	−13.57
	Cycle5	2.27	−6.63	−7.95
	Cycle6	2.45	−6.74	−8.25
Liver	Cycle1	0.32	−31.78	−41.47
	Cycle2	0.57	−29.72	−36.44
	Cycle3	0.78	−25.68	−30.25
	Cycle4	1.01	−23.44	−27.18
	Cycle5	0.95	−21.04	−24.13
	Cycle6	1.13	−21.05	−23.92
Bone marrow	Cycle1	2.82	−24.51	−56.34
	Cycle2	3.84	−18.29	−50.88
	Cycle3	4.51	−10.55	−46.06
	Cycle4	4.98	−9.12	−39.50
	Cycle5	5.82	−38.58	−50.83
	Cycle6	6.58	−40.26	−53.06
Spleen	Cycle1	0.28	−30.55	−48.80
	Cycle2	0.49	−35.90	−48.74
	Cycle3	0.63	−31.70	−40.28
	Cycle4	0.93	−31.39	−39.96
	Cycle5	1.30	−33.07	−41.92
	Cycle6	1.23	−26.71	−32.20
Tumor	Cycle1	8.50	17.38	−0.19
	Cycle2	10.71	3.61	−3.16
	Cycle3	11.73	1.98	−4.00
	Cycle4	11.52	12.59	6.21
	Cycle5	12.93	28.18	5.18
	Cycle6	18.31	36.44	10.37

$$Root\;Mean\;Squared\;Error\;\left(RMSE\right)=\sqrt{Mean\;Squared\;Error}=\sqrt{\frac1n{\sum_{i=1}^n({STPD}_i-{MTPD}_i)}^2}$$

$$Relative\;Percentage\;Difference\;(RPD)=\frac{{STPD}_i-{MTPD}_i}{\left(\frac{{STPD}_i+{MTPD}_i}2\right)}\times100$$

$$Mean\;Percentage\;Error\;\left(MPE\right)=\frac1n\sum_{i=1}^n\left(\frac{{STPD}_i-{MTPD}_i}{{MTPD}_i}\times100\right)$$

### Tumor STPD and MTPD vs therapy response

**Fig. 2:**
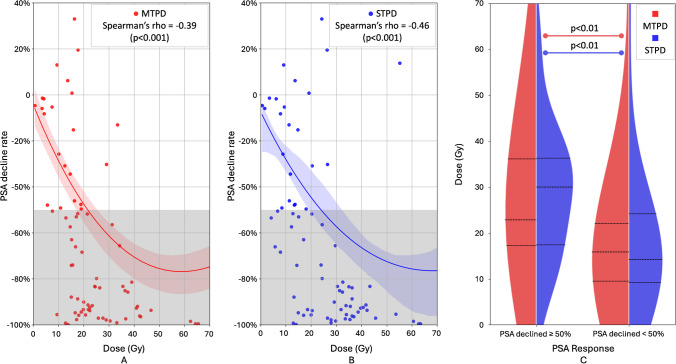
**A**, **B** Correlation between PSA declined rate with tumor (**A**) MTPD and (**B**) STPD. **C** Splited violin plot of STPD and MTPD subgrouped by PSA declined rate

Both STPD and MTPD of tumor areas showed significant correlations with the PSA decline rate (MTPD: rho = −0.39, *p* < 0.001; STPD: rho = −0.46, *p* < 0.001), as depicted in the fitted regression results (Fig. 2A, B). Fisher's Z-transformed test indicated no significant difference between the two correlations (Z = 0.58, *p* = 0.56). MTPD and STPD on tumor areas demonstrated a significant difference between subgroups with PSA decline ≥ 50% and < 50% (*p* < 0.001) according to the U-test (Fig. 2C). For patients who received more than 4 therapy cycles, the STPD_total_ (Hazard Ratio = 1.05, *p* < 0.05), log-transformed MTPD_total_ (Hazard Ratio = 3.41, *p* < 0.05), and log-transformed STPD_total_ (Hazard Ratio = 8.06, *p* < 0.05) significantly affected OS, while the MTPD_total_ was not significant. The Kaplan-Meier plots are presented in Supplemental Fig. 3A-D.

### Bone marrow STPD and MTPD vs toxicity

**Fig. 3:**
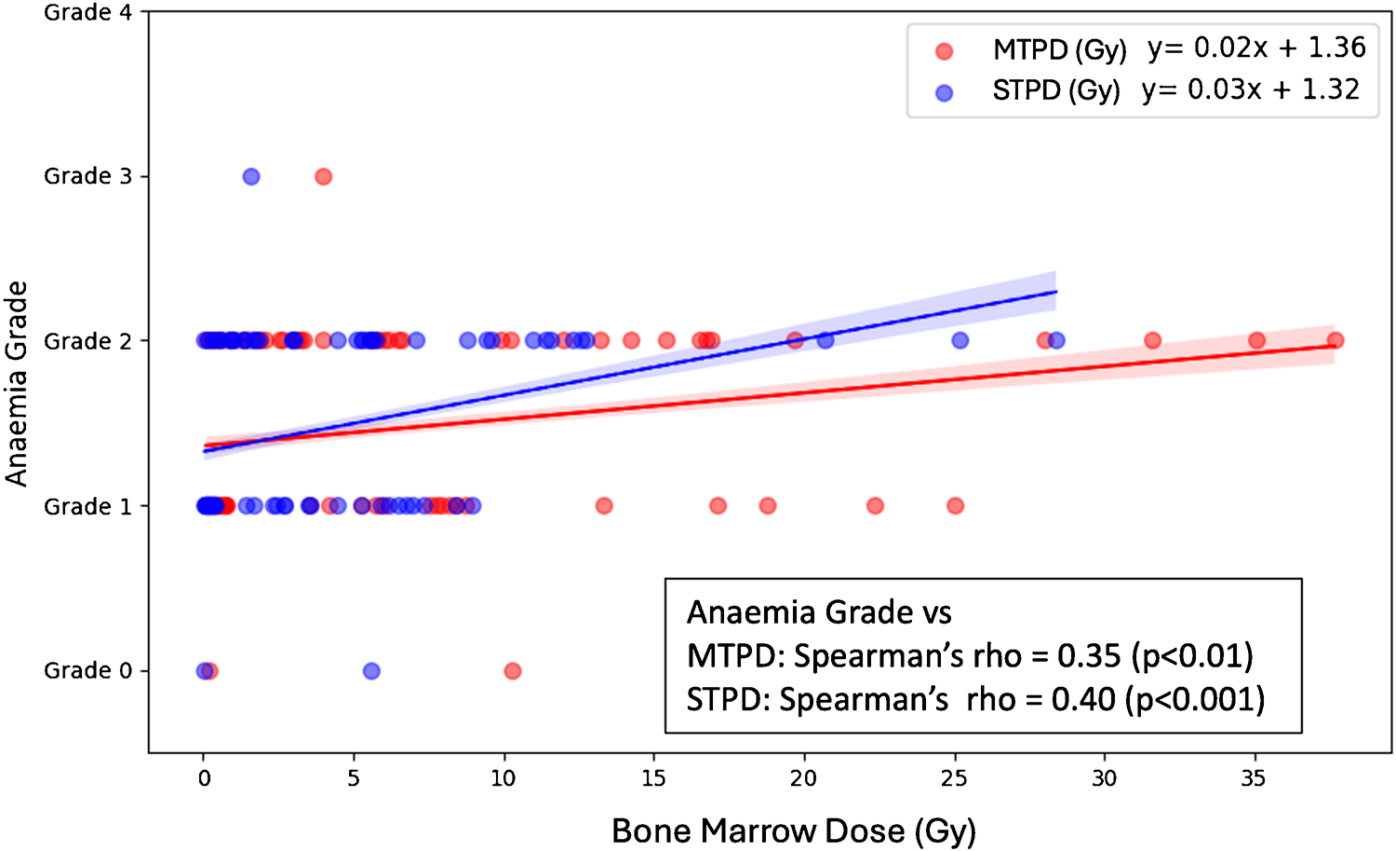
Correlation between anaemia grading with STPD and MTPD of bone marrow

The non-parametric test demonstrated a significant association between bone marrow MTPD (rho = 0.35, *p* < 0.01) and STPD (rho = 0.40, *p* < 0.001) with anaemia grades, as illustrated in Fig. 3. Fisher's Z-transformed test revealed no significant differences between the correlations of STPD and MTPD with anaemia grade (Z = −0.39, *p* = 0.70).

## Discussion

Despite advancements in dosimetry development, MTPD is currently hindered by complex logistics, additional patient burden, and resource constraints in clinical application. Furthermore, there is still limited evidence regarding dosimetry in predicting therapeutic responses and identifying potential toxicities [18, 19]. In this retrospective study, we investigated the influence of errors in a simplified dosimetry method from a clinical perspective. Here, we focused on the treatment of ^177^Lu-PSMA-617 RPT and correlated the dosimetry with follow-up data on serum PSA decline rate, toxicity over cycles, and overall survival [17].

In this study, we selected the Hänscheid STPD method as the simplified dosimetry method, which has already been applied in ^177^Lu-PSMA-617 dosimetry calculations [21, 33]. With the retrospective data, we could only select the time-points from 1–3 days post-injection for the application of the Hänscheid method, which aligns well with the Hänscheid method's recommended time window [26, 30]. For quality control, we checked the eligible time window using the population effective half-life estimated from our data to confirm the suitability of our dosimetry time-points. Nevertheless, these time-points may not represent the best performance of the Hänscheid method [21, 26, 30, 33], which could have even lower errors.

Our MTPD findings of OARs were similar to previous dosimetry studies [23, 34]. For example, in the first cycle, the kidneys’ MTPD per unit of administered activity was 0.36 ± 0.13 Gy/GBq, which is within the reference range from the VISION trial (mean: 0.43 Gy/GBq, range: 0.22–0.83 Gy/GBq) [34], and also similar to the kidneys’ dose (0.39 Gy/GBq) reported in a voxelized dosimetry study [23]. For dosimetry over 6 cycles, our results demonstrated that the kidneys’ MTPD was 17.20 ± 6.58 Gy when the mean cumulative injection activity was 39.91 ± 1.42 GBq, also within the range from the VISION trial, which is 9.1- 29 Gy when 44.4 GBq injection activity was given [34]. However, our MTPD results for bone marrow from the first cycle (0.45 ± 0.53 Gy/GBq) showed a higher dose compared to the blood sample-based dose (mean: 0.035 Gy/GBq, range: 0.02–0.13 Gy/GBq) from the VISION trial [34], and also higher than the voxelized image-based dose found in Violet’s study (mean: 0.11 Gy/GBq, range: 0.01–0.34 Gy/GBq) [23]. The gap may be related to the challenges in accurately segmenting non-tumor-infiltrated bone marrow. We utilized an automatic whole-body bone mask and subtracted the higher PSMA-PET SUV (> 50% SUV_max_) region, which is easy to perform but does not accurately delineate the marrow. The observed correlation between bone marrow dose and toxicity grading hints that both MTPD and STPD measurements could provide meaningful and relevant data for assessing the impact of radiopharmaceuticals, despite the limitations of our segmentation method. Nevertheless, our results showed a similar tumor dose (2.02 ± 1.68 Gy/GBq) compared to Steffie’s finding (all lesion median: 1.69 Gy/GBq, range: 0.41–10.34 Gy/GBq) [35].

Our results confirmed a strong agreement between STPD and the referenced MTPD for tumors and all the tested OARs (Spearman correlation’s *p* < 0.001 for all). When assessing the error of STPD relative to MTPD in our cohort, we observed an increase in RMSE across therapy cycles. This can be attributed to a combination of variances in the optimal condition of Hänscheid’s approximation [20, 21], and possible changes in metastasis composition, or treatment-induced radiobiological impacts [36, 37]. We did not find a comparison between STPD and MTPD for ^177^Lu-PSMA-617 RPT schemes over 6 cycles from previous studies. However, our results showed a larger range of error compared to previous report from the first 2 cycles, with percentage differences of the Hänscheid method compared with MTP being less than ± 20% [21]. Specifically, our results showed the largest error in bone marrow STPD, with its MPE up to 40.26% compared to MTPD. This significant error could be attributed to the spatial mismatch in the SPECT-SPECT image registration [25] and the heterogeneous red bone marrow distribution in different bone sites [38], which poses segmentation challenges in segmentation, particularly for patients with extensive bone metastases.

From a clinical perspective, the safety evaluation in our study found that renal doses from both STPD and MTPD approaches were below the established limits and consistent with clinical trials [31, 34, 39]. Furthermore, despite the great deviation between MTPD and STPD, in bone marrow we found the increasing MTPD and STPD were significantly correlated with anaemia grade (both *p* < 0.01), and no significant difference between the two corrections was found (Z = −0.39, *p* = 0.70). Additionally, the therapy response evaluation showed that both MTPD and STPD of tumor were significantly correlated with positive therapeutic outcomes (both *p* < 0.001), and there was no significant difference between the two correlations (Z = 0.56, *p* = 0.56). Note that the relationship between dose and PSA decline is observed to be nonlinear. Although we employed the Spearman’s correlation to take this nonlinearity into account, the correlation cannot fully reflect the radiobiological principles [8]. Ideally radiobiological modeling could provide more quantitative insight for the comparison, which could be future work if such kind of model can be developed [39]. Nevertheless, correlation is still the most practical and popular way in clinical assessment of the influencing factors of RPT radiobiological effects [22, 23].

This study is limited by its retrospective nature and small patient cohort. Potential biases from prior therapies, continued treatment-induced radiobiological shifts, and changes in metastasis composition during therapy cycles could impact the performance of both dosimetry methods in their associations with therapy outcomes [28, 29, 40–42]. In addition to the studied Hänscheid method, our findings need cross verification for other simplified dosimetry methods [20, 21, 26, 43–45].

Existing evidence demonstrates the potential of dosimetry in determining the treatment outcome of RPT including ^177^Lu-PSMA-617 RPT [22–24]. However, the demanding or acceptable accuracy of dosimetry measurements in clinical practice has not been well investigated, especially in practical dilemmas facing compromises with accuracy using simplified protocols. This study presents the first investigation of the influence of dosimetry errors on clinical predictive value. Despite the limitations, the preliminary findings of this study may trigger further investigation of the acceptable accuracy level of dosimetry measurements and in-depth discussion of their impact in clinical practice.

## Supplementary Information

Below is the link to the electronic supplementary material.

Supplementary file1 (DOCX 577 KB)

## Data Availability

No.
