# Supplementary material for: Influence of dosimetry accuracy on the correlation with treatment outcome in a preliminary PSMA radiopharmaceutical therapy study
Source: Eur J Nucl Med Mol Imaging. 2024 Dec 20;52(5):1649–57. doi: 10.1007/s00259-024-07010-3 (PMC11928392; doi:10.1007/s00259-024-07010-3)
Supplement: Supplementary file 1 — Supplementary file1 (DOCX 577 KB) [file 259_2024_7010_MOESM1_ESM.docx]

**Supplemental Table 1**

*Supplemental Table 1. Doses per Unit Activity per Cycle*

| Cycle | Injection Activity (GBq) | Mean Doses ± SD  (min - max)  (Gy/GBq) | Kidneys | Liver | Spleen | Bone marrow | Tumor |
| --- | --- | --- | --- | --- | --- | --- | --- |
| 1st | 7.21 ± 0.28  (6.60 - 7.40) | MTPD-based | 0.36 ± 0.13  (0.07 - 0.67) | 0.13 ± 0.07  (0.04 - 0.30) | 0.09 ± 0.07  (0.01 - 0.36) | 0.45 ± 0.53  (0.02 - 1.80) | 2.02 ± 1.68  (0.08 - 6.19) |
|  |  | STPD-based | 0.29 ± 0.12  (0.05 - 0.57) | 0.09 ± 0.08  (0.03 - 0.32) | 0.05 ± 0.06  (0.02 - 0.27) | 0.25 ± 0.27  (0.02 - 1.80) | 2.00 ± 1.54  (0.08 – 6.00) |
| 2nd | 6.90 ± 0.49  (5.90 - 7.50) | MTPD-based | 0.48 ± 0.24  (0.11 - 1.19) | 0.13 ± 0.06  (0.04 - 0.26) | 0.09 ± 0.03  (0.04 - 0.17) | 0.29 ± 0.39  (0.01 - 1.45) | 1.56 ± 1.82  (0.09 - 7.76) |
|  |  | STPD-based | 0.41 ± 0.18  (0.2 - 0.87) | 0.09 ± 0.03  (0.03 - 0.18) | 0.05 ± 0.03  (0.03 - 0.13) | 0.20 ± 0.24  (0.01 - 0.82) | 1.10 ± 1.02  (0.17 - 3.81) |
| 3rd | 7.02 ± 0.37  (6.00 - 7.40) | MTPD-based | 0.48 ± 0.21  (0.24 - 0.99) | 0.13 ± 0.06  (0.08 - 0.28) | 0.08 ± 0.04  (0.04 - 0.18) | 0.25 ± 0.45  (0.01 - 1.75) | 0.67 ± 0.90  (0.18 - 3.80) |
|  |  | STPD-based | 0.41 ± 0.21  (0.17 - 0.98) | 0.11 ± 0.05  (0.06 - 0.20) | 0.06 ± 0.04  (0.02 - 0.13) | 0.17 ± 0.36  (0.01 - 1.43) | 0.69 ± 0.88  (0.04 - 3.68) |
| 4th | 6.66 ± 1.03  (3.70 - 7.50) | MTPD-based | 0.43 ± 0.11  (0.27 - 0.59) | 0.15 ± 0.12  (0.08 - 0.56) | 0.11 ± 0.04  (0.05 - 0.20) | 0.14 ± 0.19  (0.01 - 0.58) | 0.48 ± 0.43  (0.15 - 1.45) |
|  |  | STPD-based | 0.44 ± 0.19  (0.21 - 0.85) | 0.12 ± 0.10  (0.06 - 0.45) | 0.07 ± 0.03  (0.03 - 0.15) | 0.11 ± 0.20  (0 - 0.75) | 0.71 ± 0.89  (0.10 - 2.97) |
| 5th | 6.71 ± 0.67  (5.90 - 7.50) | MTPD-based | 0.38 ± 0.14  (0.19 - 0.65) | 0.11 ± 0.03  (0.09 - 0.17) | 0.11 ± 0.07  (0.05 - 0.28) | 0.1 ± 0.15  (0.01 - 0.43) | 0.39 ± 0.34  (0.13 - 1.29) |
|  |  | STPD-based | 0.39 ± 0.15  (0.18 - 0.60) | 0.09 ± 0.03  (0.05 - 0.13) | 0.06 ± 0.04  (0.02 - 0.16) | 0.08 ± 0.15  (0 - 0.53) | 0.50 ± 0.49  (0.14 - 1.55) |
| 6th | 5.37 ± 1.11  (3.70 - 6.10) | MTPD-based | 0.42 ± 0.20  (0.27 - 0.8) | 0.11 ± 0.03  (0.07 - 0.18) | 0.09 ± 0.03  (0.06 - 0.13) | 0.18 ± 0.29  (0.01 - 0.80) | 0.36 ± 0.27  (0.09 - 0.92) |
|  |  | STPD-based | 0.40 ± 0.18  (0.16 - 0.68) | 0.09 ± 0.03  (0.06 - 0.14) | 0.07 ± 0.02  (0.05 - 0.11) | 0.14 ± 0.25  (0.01 - 0.70) | 1.83 ± 4.22  (0.10 - 11.39) |

**Supplemental Figure 1**


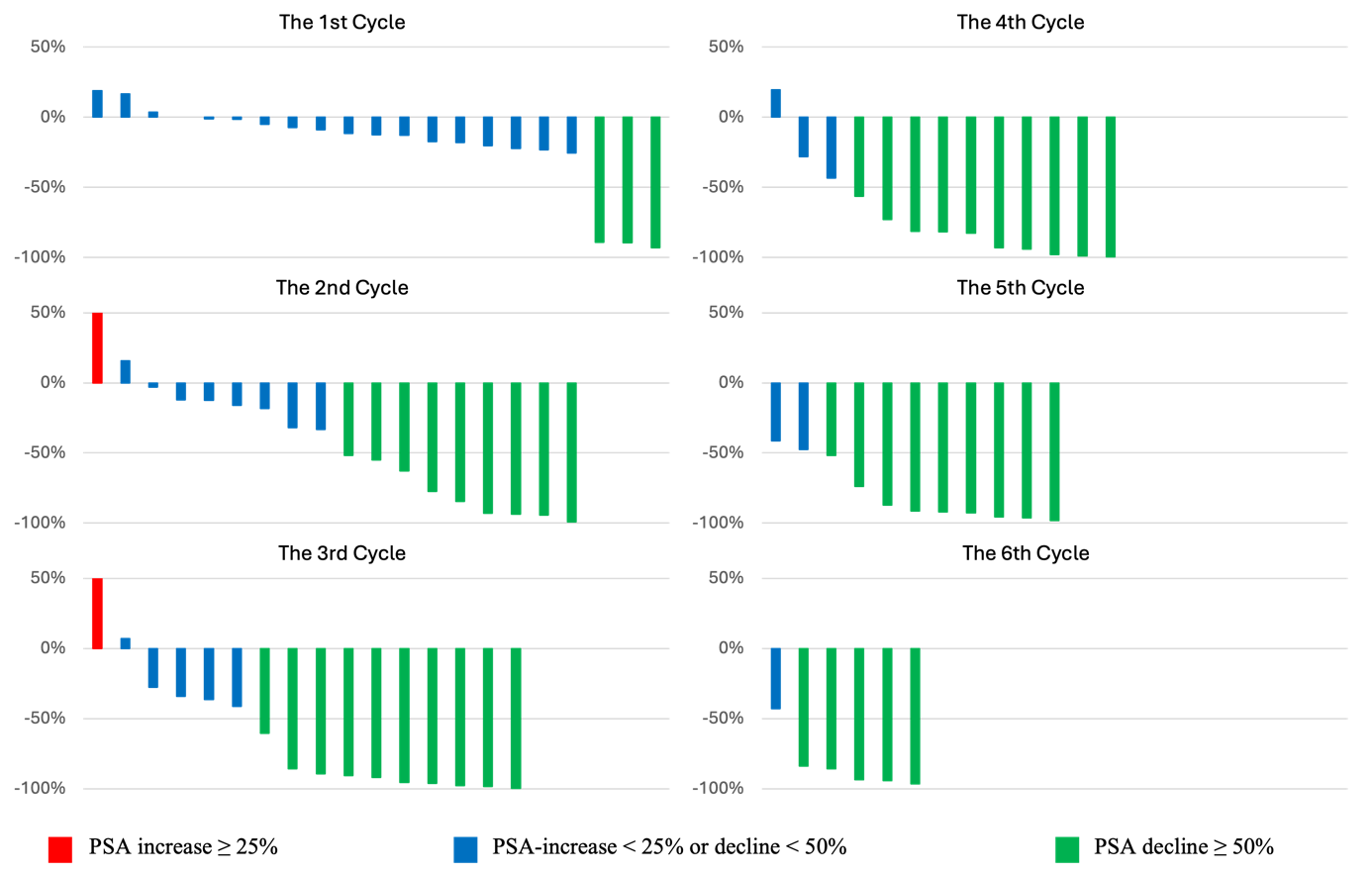


*Supplemental Figure 1. PSA response following ^177^Lu-PSMA-617 RPT treatment (waterfall plots)*

*The biochemical response was classified according to the recommendations of the Prostate Cancer Working Group 3 (PCWG3):*

*- Partial response (PR) if PSA-decrease≥50%*

*- Stable disease if PSA-increase <25% and PSA-decrease <50%*

*- Progressive disease (PD) if PSA-increase≥25%*

**Supplemental Figure 2**

*Supplemental Figure 2. Correlation between MTPD (Gy)* *and STPD (Gy)*


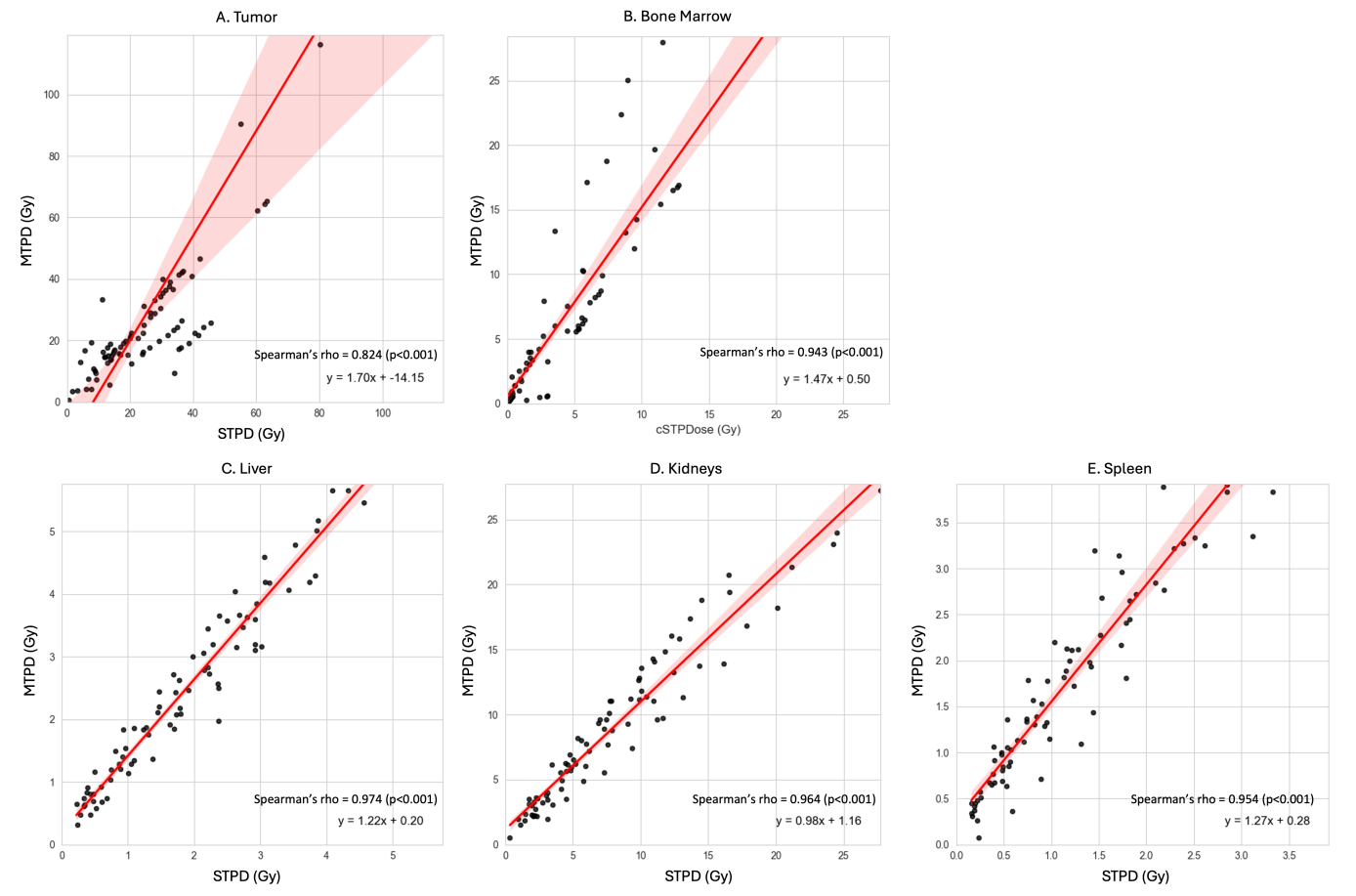


**Supplemental Figure 3**

*Supplemental Figure 3.*

*A-D. Correlation between* *OS time with total tumor dose: (A) MTPD_total_,* *(B) STPD_total_, (C) log-transformed MTPD_total_ (log_ MTPD_total_), and (D) log-transformed STPD_total_ (log_STPD_total_).*

*
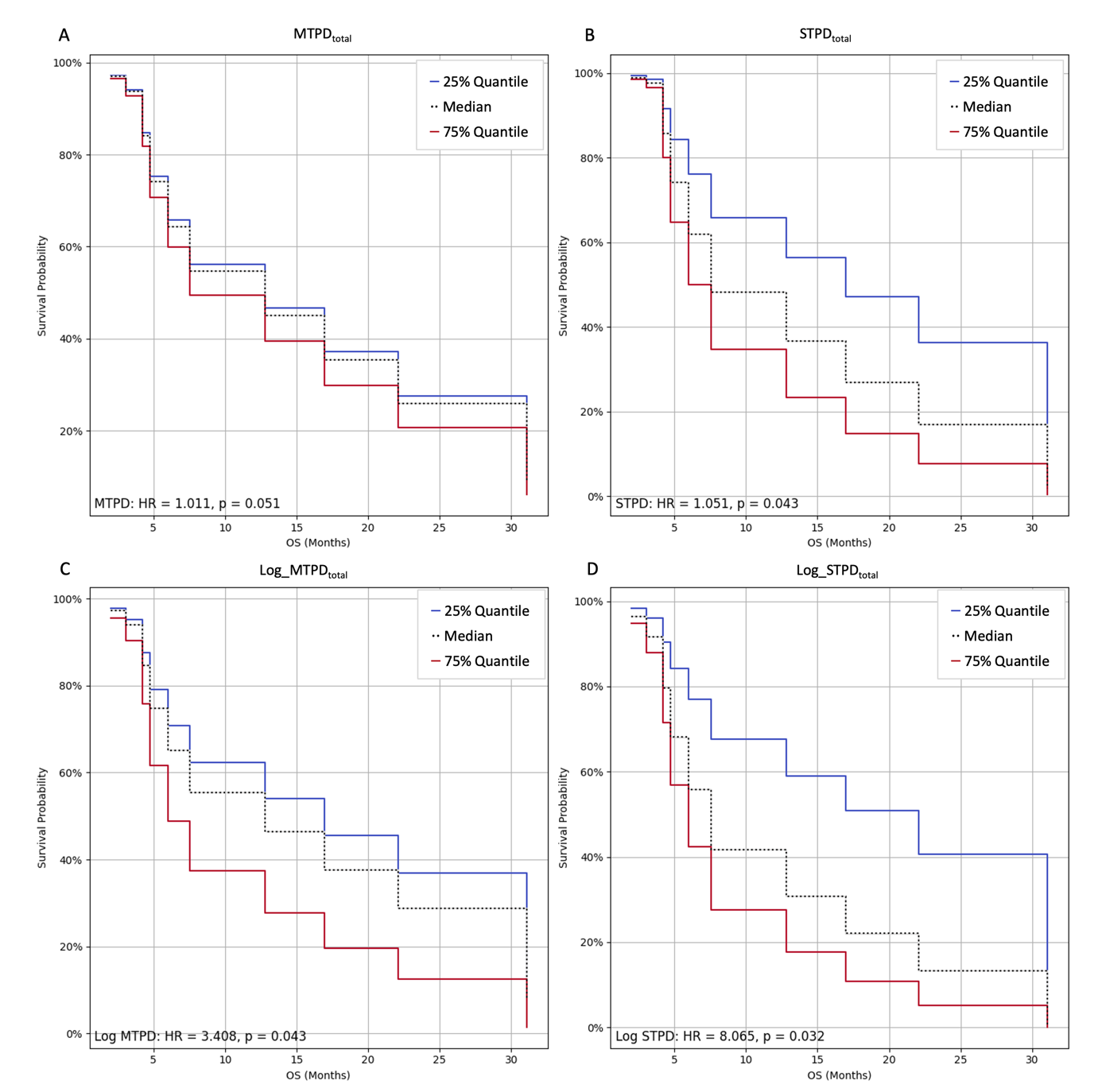
*
